# Targeting the Notch Signaling Pathway in Chronic Inflammatory Diseases

**DOI:** 10.3389/fimmu.2021.668207

**Published:** 2021-04-12

**Authors:** Panagiotis F. Christopoulos, Torleif T. Gjølberg, Stig Krüger, Guttorm Haraldsen, Jan Terje Andersen, Eirik Sundlisæter

**Affiliations:** ^1^ Department of Pathology, University of Oslo and Oslo University Hospital, Oslo, Norway; ^2^ Institute of Clinical Medicine and Department of Pharmacology, University of Oslo and Oslo University Hospital, Oslo, Norway; ^3^ Centre for Eye Research and Department of Ophthalmology, University of Oslo and Oslo University Hospital, Oslo, Norway; ^4^ Department of Immunology, University of Oslo and Oslo University Hospital, Oslo, Norway

**Keywords:** notch, inflammation, autoimmunity, gamma-secretase inhibitors (GSIs), neutralizing antibodies, biological therapeutics

## Abstract

The Notch signaling pathway regulates developmental cell-fate decisions and has recently also been linked to inflammatory diseases. Although therapies targeting Notch signaling in inflammation in theory are attractive, their design and implementation have proven difficult, at least partly due to the broad involvement of Notch signaling in regenerative and homeostatic processes. In this review, we summarize the supporting role of Notch signaling in various inflammation-driven diseases, and highlight efforts to intervene with this pathway by targeting Notch ligands and/or receptors with distinct therapeutic strategies, including antibody designs. We discuss this in light of lessons learned from Notch targeting in cancer treatment. Finally, we elaborate on the impact of individual Notch members in inflammation, which may lay the foundation for development of therapeutic strategies in chronic inflammatory diseases.

## Introduction

The Notch signaling pathway is a major regulator of cell-fate determination during development and cellular differentiation (1, 2). It was identified more than 100 years ago in *Drosophila melanogaster*, in which haploinsufficiency of the Notch locus and the associated partial loss of Notch function gave rise to a notched wing (3). Unlike soluble ligands, like tumor necrosis factor (TNF) and interleukin (IL) 1 beta, triggering conventional inflammatory receptor signaling, Notch ligands are, like their receptors, transmembrane proteins with large extracellular domains. Therefore, Notch signaling represents a short-range signaling system for direct cell-to-cell communication.

Emerging evidence points to Notch signaling as a modulator of innate and adaptive immune responses (4), and manipulating the pathway has been suggested as an attractive therapeutic strategy to target immunological diseases (5, 6). Numerous reports have provided insights into the outcome of manipulating Notch signaling in experimentally induced inflammation by a range of genetic and pharmacological strategies, including lymphoid- or myeloid-lineage-specific deletion of components of the pathway, systemic pan-Notch inhibition, and selective blockade of individual Notch ligands and receptors (7–10). Although the studies have revealed conflicting roles for Notch in the development, differentiation, and activation of different immune cell subsets, the collective data strongly indicates that Notch activation in most cell types supports a pro-inflammatory phenotype, and that pharmacologic inhibition of this signaling cascade may hold therapeutic potentials in several inflammatory diseases.

In this review, we summarize the emerging roles of Notch signaling in regulating immune responses and highlight strategies that may be used to target the Notch pathway in chronic inflammation. From a disease-oriented standpoint, we focus on classical autoimmune diseases such as rheumatoid arthritis, inflammatory bowel disease and uveitis, and also discuss recent findings from preclinical models of graft-versus-host disease. To date, cancer has been the main focus area of potential therapeutic strategies, for both selective Notch modulation and pan-Notch inhibition, generating an accumulating body of evidence regarding possible disadvantages and side-effects that must be considered to tailor design of future treatment options. Finally, we briefly summarize lessons learned in tumor models, discuss potential therapeutic strategies for Notch modulation in chronic inflammation and suggest improvements in the field.

### The Notch Signaling Pathway**

**Figure 1:**
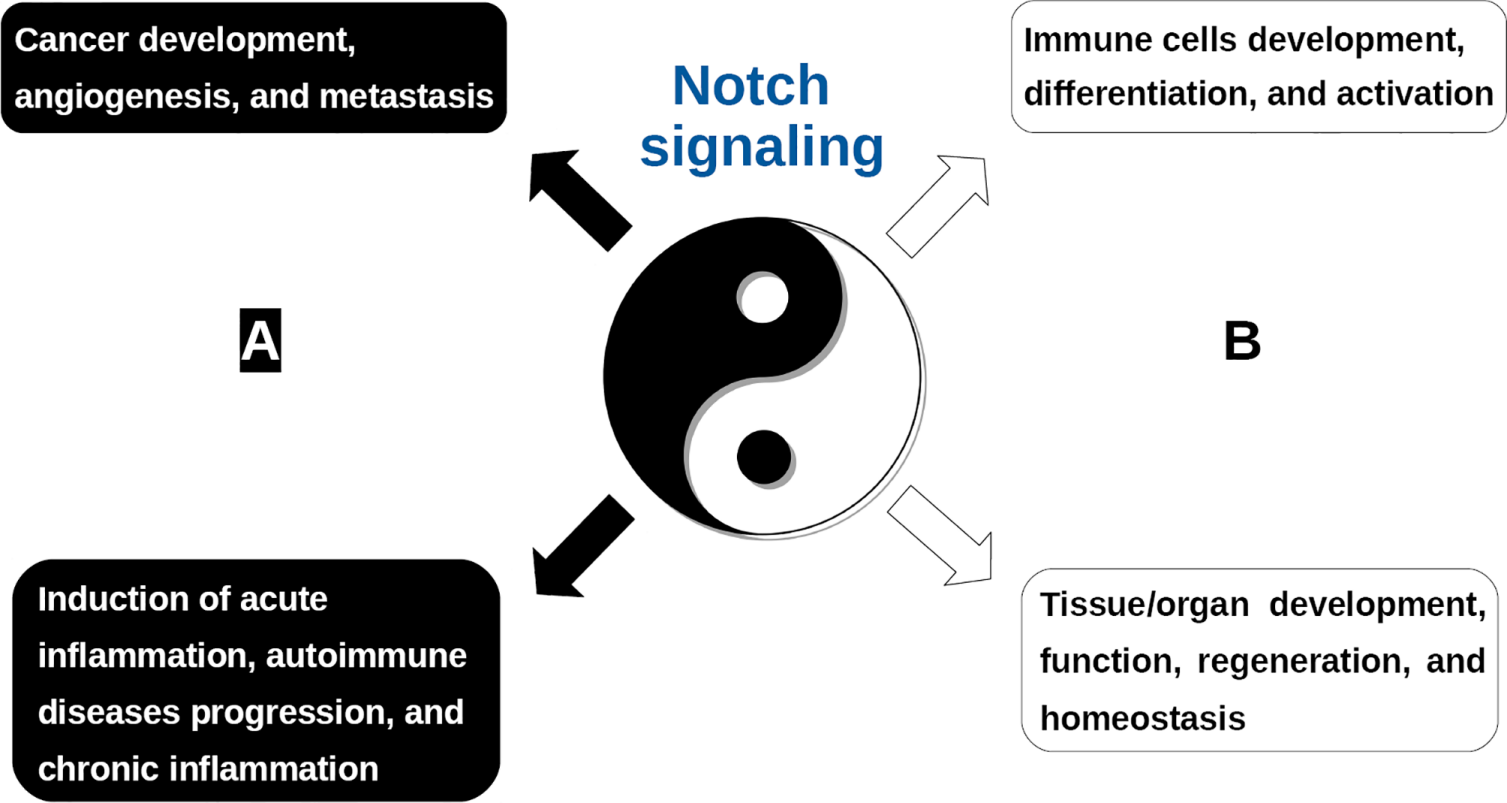
The “yin and yang” of the Notch signaling. As with most major signaling pathways, the Notch system is implicated in both; **(A)** pathological processes (yin; indicated in black frame), including, but not limited to, cancer progression and metastasis, as well as, autoimmune diseases, acute and chronic inflammation and **(B)** favorable homeostatic processes (yang; indicated in white frame), including, but not limited to, organ development and function, as well as, immune cells differentiation and activation.

**Figure 2:**
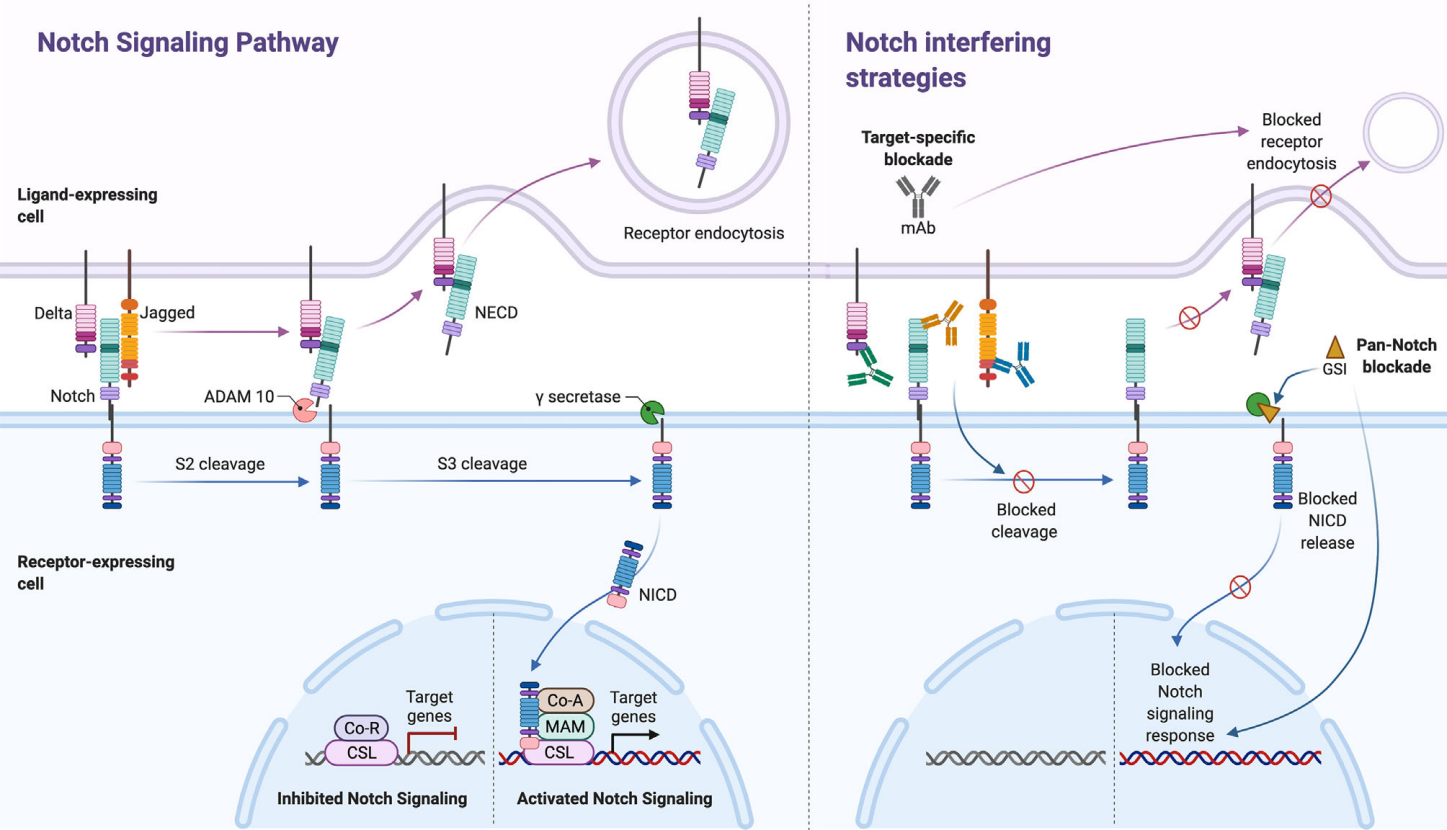
The Notch signaling pathway and its inhibitors. Notch signaling (left panel, separated by stapled line) is activated upon ligand (Delta or Jagged) binding to an adjacent Notch receptor on a neighboring cell. Upon activation, Notch receptors are cleaved, first by ADAM, in the NRR of the extracellular domain (S2 cleavage). The released extracellular domain (NECD) is then trans-endocytosed by the ligand-expressing cell. The second cleavage (S3 cleavage) is mediated by the γ-secretase (GS) complex, which releases the Notch intracellular domain (NICD) into the cytoplasm, thus allowing its activation and nuclear translocation. In the nucleus, NICD binds to the transcription factor CSL family proteins and the Mastermind (MAM)-like transcriptional co-activator 1. The assemble of this transcription activation complex on the recombination signal-binding protein for immunoglobulin kappa J (RBP-J) promoter region, leads to the expression of various downstream target genes. Two main Notch-interfering strategies (right panel) exists; monoclonal antibodies (mAbs) against the ligands and/or receptors (target-specific blockade) and GS inhibitors (GSI, pan-Notch blockade). While mAbs may block specific ligand-induced S2 cleavage and consecutively trans-endocytosis of NECD, GSI strategies will non-specifically block NICD release following all ligand-receptor interactions. The figure was made in BioRender (https://biorender.com).

Notch signaling is mediated by a highly conserved ligand-receptor apparatus and plays a key role in the regulation of cellular proliferation, survival, apoptosis, and differentiation, thereby critically affecting organ development and function (11, 12). However, as with most signaling pathways in human pathophysiology, it is also implicated in the development and progression of various human cancers and autoimmune diseases (**Figure 1**). Notch genes encode single-pass transmembrane receptors, activated by a dual proteolytic cleavage, which releases the Notch intracellular domain (NICD), allowing its nuclear translocation. Notch ligand binding mediates receptor activation by instigating a conformational change required for such cleavage to take place. In the nucleus, NICD binds to the transcription factor CSL family proteins [recombination signal-binding protein 1 for J-kappa (RBP-Jk) in mammals], and co-activators like the Mastermind-like transcriptional co-activator 1 (MAML-1). The assemble of this transcription activation complex leads to the transcription of various downstream target genes (**Figure 2**, left panel). To date, four genes encoding Notch receptors (Notch 1-4) have been identified in mammals, including humans. In addition, five genes encoding Notch receptor ligands have been reported. The mammalian ligands are classified on the basis of structural homology to the two Drosophila ligands, Delta and Serrate, and designated as either Delta-like (DLL1, -3, and -4) or as Serrate-like, known as Jagged in mammals (Jagged-1 and Jagged-2).

The four Notch receptors share similarities in their structural architecture, with distinct subtle differences in their extracellular and intracellular domains (13). Following proteolytic cleavage in the secretory pathway, the receptors form a heterodimer of which the extracellular domain is non-covalently bound to the transmembrane-intracellular domain. The extracellular domain of Notch receptors contains up to 36 repeated copies of an epidermal growth factor (EGF)-like motif, which are involved in ligand interaction. These amino-terminal EGF-like repeats are followed by a negative regulatory region (NRR), which contains three cysteine-rich Lin12-Notch repeats (LNR) and a heterodimerization domain (HD), and together, these function to inhibit Notch activation in the absence of ligands. The NICD contains a RBP-Jk-association molecule (RAM) module, ankyrin (ANK) repeats, nuclear localization signals (NLS), a trans-activation domain (TAD), and a negative regulatory peptide sequence, rich in proline, glutamine, serine and threonine residues (PEST). The RAM and ANK domains enable docking of the NICD to other proteins while the PEST sequence is involved in Notch protein turnover (12).

Notch ligands also have multiple EGF-like repeats in their extracellular domain. The Serrate ligands Jagged-1 and Jagged-2 have almost two-fold as many EGF-like repeats compared to Delta, and, in addition, feature a cysteine-rich region (CR) that is absent in Delta.

Notch signaling is activated upon ligand binding to an adjacent Notch receptor on a neighboring cell. In contrast, receptor association with ligands presented by the receptor-bearing cell (*cis*-presented ligand) inhibit receptor activation (14). Upon activation, Notch receptors are cleaved twice. The first step is mediated by ADAM17 (a disintegrin and metalloprotease) or ADAM10 (15), which cleaves the receptor at the extracellular S2 cleavage site (Site 2), situated deep in the NRR in the posed receptor. Ligand binding in *trans* causes a pulling force, which opens up the NRR and exposes the S2, thus allowing this cleavage to initiate the activation process. The released extracellular domain is then trans-endocytosed by the ligand-expressing cell. The second cleavage, mediated by the γ-secretase complex (comprised of presenilin-1/2, nicastrin, Pen-2, and Aph-1), releases NICD into the cytoplasm, thus allowing its nuclear translocation (**Figure 2**, left panel). This sequence has become known as the canonical Notch pathway.

There is also some evidence to support the existence of non-canonical Notch signaling [reviewed in (16, 17)]. Type I non-canonical signaling involves Notch activation and transfer of activation signals independent of RBP-Jk (NICD-dependent but RBP-Jk-independent) (18, 19); Type II involves activation of Notch target genes independent of proteolytic cleavage of Notch and RBP-Jk activation (NICD- and RBP-Jk-independent) (20); Type III involves RBP-Jk-dependent gene activation without receptor cleavage and NICD release (21, 22). Several signaling pathways might be involved, such as Wnt, NfkB, HIF and PI3K/AKT. In conclusion, data on non-canonical Notch signaling is limited, and it is less characterized than the canonical pathway.

### Notch Signaling in Immune Cell Development, Differentiation, and Activation

In hematopoiesis, the best-studied functions of Notch signaling are its roles during lymphocyte development, in particular during T cell lineage commitment and maturation in the thymus, and during marginal zone B cell development in the spleen [reviewed in (4, 23)]. After common lymphoid progenitors have migrated from the bone marrow to the thymus, thymic epithelial cells expressing DLL4 trigger canonical Notch1 signaling in early thymic progenitors (24–26). DLL4-Notch1 signaling is essential for T cell lineage commitment and is further required during early phases of thymocyte differentiation. Notch signaling also inhibits other lineage potentials, such as B cell and myeloid cell potential (27). Furthermore, after newly formed B cells migrate from bone marrow to the spleen, Notch2 activation by DLL1 is required to generate the marginal zone B cells (28, 29).

However, reports on Notch functions in T helper cell polarization are contradictory, as it surfaced as a potential dictator of mutually exclusive subtypes depending on the experimental context; Notch requirement has been reported for Th1 (30), Th17 (31) and Th2 (32) polarization, as well as in Treg polarization (33). This has led to the proposed theory of Notch as an unbiased amplifier of polarizing signals, like cytokines, whose requirement was not instructive in the T helper polarization process (34). The original instructive theory of Notch was based on the observation that expression of Jagged or Delta-like ligands on antigen-presenting cells (APCs), e.g., dendritic cells, varied depending on the identity of the activating stimulus (7). Dictated by the ligand expressed on APCs, the conveyed Notch signal in approaching T-cells could thus be perceived differently, and subsequently instruct polarization in a ligand-specific manner. The unbiased amplifier model is, however, intuitively a more unifying explanation, suggesting that Notch simultaneously reinforce Th1, Th2 and Th17 specific transcriptional programs irrespective of the cytokine stimuli. In this way, Notch could allow - rather than direct – polarization. Instructive functions of Notch in T helper cell polarization may still be true in some contexts, such as at defined stages of differentiation. Also, there are reports of ligand-independent functions of Notch upon T-cell receptor activation (35). In addition, Notch is also required for cytotoxic (CD8+) T-cell effector differentiation (36), as well as T-cell stimulating processes, such as increased production of the T-cell proliferating factor IL2 (37).

Furthermore, loss-of-function studies have revealed that Notch signaling is playing important roles in innate immunity. One example is Notch 2 signaling in dendritic cell differentiation and homeostasis in the spleen and intestine (38, 39). Notch signaling also seems to be involved in promoting the pro-inflammatory activation of primary human blood monocyte-derived macrophages, where pro-inflammatory stimuli, such as lipopolysaccharide (LPS), induced expression of DLL4 in a Toll-like receptor 4 (TLR4)-NfkB dependent manner (40). Interestingly, exposure of macrophages to DLL4 induced inflammatory nitric oxide synthase (iNOS) (40), a key regulator in the classical (pro-inflammatory) activation of murine macrophages, according to the traditional definitions of macrophages polarization (41, 42). Of note, the “don’t eat me” signal axis CD47-SIRPa, currently under clinical investigation for antibody-based blocking in cancer trials (43), may also be affected by Notch signaling – Notch negatively regulates downstream effectors (Src homology region 2 domain-containing phosphatase-1, SHP-1) of the axis, and pan-Notch inhibition through a γ-secretase inhibitor (GSI) reduced the phagocytic properties of mouse (C57BL/6) bone marrow-derived macrophages (44). Of note, recent evidence points to the tissue-imprinting role of Notch signaling in the establishment and maintenance of liver-resident macrophages (Kupffer cells). Direct interaction of recruited monocytes with DLL4 on sunisoidal endothelial cells was shown to be a critical step in the re-population of depleted Kupffer cells (45, 46), highlighting the role of Notch in monocyte engraftment following liver infection or injury and the acquirement of a tissue-resident macrophage phenotype. Moreover, the development of innate lymphoid cells (ILCs), which may have a key role in regulating immunity, inflammation as well as tissue repair in multiple anatomical compartments (47), has also been reported to be influenced by Notch signaling (48).

Thus, the Notch signaling pathway plays a regulatory role in the differentiation and activation of a range of immune cell types. Although conflicting evidence exists, results support that Notch activation in most of these cell types triggers a pro-inflammatory phenotype. **Table 1** gives an overview of the role of Notch in various immune cell subsets.

**Table 1:** The Notch signaling in immune cell differentiation and activation.

Cell type	Reported action(s)	Model system(s)	References *(PMID)*
	**Adaptive immune system**		
**T helper cells (Th)**	DLL1 on antigen-presenting cells (APCs) promoted differentiation of naïve T cells into Th1 effector cells, while Jagged1 instructed naïve T cells to differentiate into the Th2 lineage.	T cells from various transgenic mice	*15137944*
	Notch1 and 2 were shown to be required on CD4+ T cells for physiological Th2 responses to parasite antigens, and GATA3 was necessary for Notch-induced Th2 differentiation.	RBPJ-deficient mice immunized with extract of eggs from *Schistosoma mansoni*	*17658279*
	Interactions between DLL4 on antigen-presenting cells (APCs) and Notch on naïve T cells *in vivo* were critical in determining the magnitude and quality of primary immune responses. The frequency of CD4+ T cells that became activated and secreted IL-2 in response to a given concentration of antigen was greater when T cells were primed in the presence of DLL4.	Mice in which DLL4 was conditionally deleted in DCs.	*25607460*
	Treatment with pan-Notch inhibitors reduced Th17-mediated disease progression in several experimental mouse models of autoimmune disease.	Experimental autoimmune encephalomyelitis, collagen-induced arthritis, OVA-induced asthma, humanized mouse model of vasculitis	*21685328 24492199 26339131 21220737*
**Cytotoxic T lymphocytes (CD8^+^ T)**	Inhibition of Notch signaling in CD8+ T cells using GSI reversed their effects on allergen-induced hyperresponsiveness and airway inflammation.	Allergen-induced airway hyperresponsiveness	*18426985*
**Regulatory T cells (T_regs_)**	Treg-specific loss of Notch function protected mice from graft-versus-host disease (GVHD).	GVHD mouse models.	*26437242*
	Inhibition of Notch1, Notch2, DLL1 or DLL4 promoted tolerance in mouse models of GVHD in association with the expansion of Tregs. Neutralizing DLL4 with a monoclonal antibody increased the pool of CD4+Foxp3+ Tregs and decreased the severity of disease.	Experimental autoimmune encephalomyelitis.	*23454750 23634056* *21813770*
**B cells**	Notch was able to synergize with the B-cell receptor and/or CD40 signaling to enhance some aspects of B-cell activation and function.	Mice in which MAML1 was conditionally deleted in follicular B cells.	*17179224*
	Lack of RBP-J caused no defects in B cells maintenance, survival, plasma cell differentiation or activation. Mice with RBP-J-deficient B cells had no obvious changes in immune responses to various antigens.	Mice in which RBP-J was conditionally deleted in B cells and then immunized with LPS, Ficoll or chicken gammaglobulin.	*11967543*
	Notch1 deficiency in murine primary B cells significantly decreased B-cell activation and antibody secretion under the presence of Notch ligand.	Murine primary B cells in which Notch1 was conditionally deleted.	*24913005*
	**Innate immune system**		
**Macrophages**	Notch1-RBP-J controlled the expression of TLR4-induced inflammatory molecules in bone marrow derived macrophages and promoted host defense against the intracellular pathogen *Listeria monocytogenes*.	Mice with myeloid-specific deletion of RBP-J infected with *Listeria monocytogenes*.	*22610140*
	Notch1 activation promotes reprogramming of mitochondrial metabolism to augment pro-inflammatory macrophage polarization in primary murine macrophages and macrophages cell line. Conditional Notch1 deletion *in vivo* attenuated the pro-inflammatory polarization of hepatic macrophages and liver inflammation.	Pharmacologic (DAPT) or genetic inhibition (myeloid-specific Notch1 KO mice) of Notch in mouse models of alcoholic steatohepatitis or galactosamine/LPS-induced fulminant hepatitis.	*25798621*
	Notch signaling plays a key role in the re-population of Kupffer cells via interaction of recruited monocytes with DLL4 on sunisoidal endothelial cells.	Kupffer cell depletion in mice models specifically expressing the diphtheria toxin (DT) receptor in Kupffer cells	*31561945* *31587991*
**Natural killer cells (NK)**	Stimulation of NK cells with Jagged2 was shown to strengthen mature NK cell-killing activity *in vivo*, and Jagged2 expressed on dendritic cells plays a crucial role in dendritic cell-mediated NK cell activation through interaction with Notch.	BALB/c or SCID mice inoculated with B-cell lymphoma cells	*18458347*

### Rationale for Therapeutic Targeting of the Notch Pathway

Given the distinct features of Notch signaling, targeting this pathway may open new doors for treatment of a range of diseases. While other signaling pathways rely on enzymatic signal amplification, Notch signaling does not, but rather relies on stoichiometric interactions between elements of the pathway (49). Thus, signal intensity can be modulated precisely by cellular regulatory mechanisms. Although there are examples of heterogeneous phenotypic responses to NICD overexpression (50), the downstream effects of Notch activation are in general dose dependent. This implies that complete shutdown of the pathway may not be necessary to achieve a therapeutic effect (51).

Another key feature is that the half-life of the active form of Notch in the nucleus is short, which is important for the dynamic control of Notch signaling (52). As such, Notch signal is a transient, short pulse of gene regulation (53). This implies that sustained inhibition may not always be necessary and that intermittent inhibition can be sufficient to obtain a therapeutic benefit.

Importantly, the effects of Notch may be context dependent. This means that Notch signals trigger distinct responses in different cell types at different time points, and that systemic inhibition of Notch signaling is likely to have a number of effects in a variety of tissues. Thus, for therapeutic utility it will be mandatory to determine whether there is a level and timing of Notch inhibition that is sufficient to attain efficacy in disease control without causing intolerable adverse side-effects. Additional challenges in this context relate to unexplored, but likely roles of different Notch receptors and ligands and their combinations, as specific signaling outcomes might underline particular ligand-receptor pairs. Moreover, all Notch ligands and receptors are transmembrane proteins with extracellular domains that are required for binding and receptor activation, making them reachable for circulating therapeutics. Combined with increasing evidence for aberrant regulation of Notch signaling in several inflammatory disorders, this makes Notch ligands and receptors possibly attractive therapeutic targets. To date, the two main strategies targeting Notch (**Figure 2**, right panel) are 1) GSIs that block the release of active NCID and 2) monoclonal antibodies (mAbs) that either block ligation upon cell contact or stabilize the NRR-region to prevent proteolytic cleavage.

#### γ-Secretase Inhibition

GSIs were originally developed to block the production of amyloidogenic peptides in Alzheimer’s disease, which also relies on γ-secretase. This multi-subunit enzyme complex targets about 100 transmembrane proteins (54), including the amyloid precursor protein, involved in this neurodegenerative disease. Inhibiting gamma-secretase function prevents the cleavage of the Notch receptors and block Notch signal transduction. Thus, GSIs, such as, DAPT (N-[N-(3,5-Difluorophenacetyl)-L-alanyl]-S-phenylglycine t-butyl ester) or DBZ (dibenzazepine) could pave the way for treatment of diseases where the Notch signaling pathway is central in the pathogenesis and/or progression of disease. Consistent with this rationale, several clinical trials of GSIs in cancer have been conducted (55). The first clinical trial with a GSI Notch inhibitor (MK-0752) was performed for more than 10 years ago on patients with T cell acute lymphoblastic leukemia/lymphoma (T-ALL) (NCT00100152). While deemed optimistic at the time, follow-up studies for T-ALL have not been launched (https://www.clinicaltrials.gov/ct2/results?term=MK-0752&draw=1&rank=9#rowId8). However, MK-0752 has been investigated against refractory central nervous system cancer, pancreatic cancer as well as both metastatic and early-stage breast cancer. Notably, 3 out of these 9 clinical trials have been terminated and follow-up studies of the remaining 6 have yet to be announced. Serious adverse events (SAEs) are common, for instance occurring in 16 out of 30 participants in a 2014 study of metastatic breast cancer (NCT00645333). One of the first and most prominent SAE from Notch inhibition is severe diarrhea. This is most likely due to on-target Notch1 inhibition in intestinal stem cells, resulting in excessive numbers of mucus-secreting goblet cells and inhibition of epithelial cell division (56). Diarrhea has been observed as a dose-limiting toxicity in several clinical trials stratifying GSIs (57, 58), and will likely be the downfall of any systemically delivered pan-Notch inhibitor. Notably, intermittent dosing of a GSI, improved gastrointestinal toxicity to tolerable levels in patients with advanced solid tumors (57).

A phase 3 trial of the GSI semagacestat (NCT00594568), also developed initially for treatment of Alzheimer’s disease, was terminated before completion due to lack of clinical efficacy and safety concerns (59). Adverse events in this case included infections, skin reactions and skin cancers, suggesting on-target Notch inhibition-related events. In line with this, laboratory findings included alterations of immune-cell populations, such as reduced lymphocyte counts, and reduction in Immunoglobulin G (IgG) levels. Despite that the immunosuppressive role of Notch inhibition is well established, its precise roles and effects on the various immune cell subsets are not yet clear. Thus, increased infections in patients treated with semagacestat is not surprising given the significant role of Notch signaling in the activation and differentiation of immune cells serving as the defense line against pathogenic bacteria, viruses and fungi. The increased rate of skin cancer is underpinned by loss-of-function experiments demonstrating a tumor suppressor function of Notch signaling in both mice and human keratinocytes (60–62). Notably, loss of Notch signaling in the murine skin led to atopic dermatitis-like disease and associated myeloproliferative disorder, demonstrating a key role for Notch in homeostatic regulation of the skin and the hematopoietic system (63).

Furthermore, while never reported as an adverse event in humans, administration of a GSI (LY 411575, 5 mg/kg per day for 3 months) in mice resulted in morphologic changes of the liver in the form of minor periportal lymphocytic infiltration (33). The authors hypothesized that Notch inhibition blocked TGFβ-mediated Foxp3 expression, resulting in defective ability to suppress naive T-cell proliferation. These findings are, however, in contrast to a recent study showing that Notch signaling has inhibitory effects on Treg cell function and that Treg cell-specific loss of Notch function actually protects mice from graft-versus-host disease (GVHD) (64). Nevertheless, one cannot rule out that long-term systemic pan-Notch inhibition is the primary causal factor for these morphological changes, since examination of livers from Notch1 antisense mice, which have had reduced Notch1 signaling throughout their entire life span, revealed a more striking infiltration (33). Thus, patients treated long-term with GSIs should most likely be monitored using liver function tests and ultrasound liver imaging.

In addition, hair graying can also be caused by systemic Notch inhibition (65), as conditional deletion of Notch1 and Notch2 in the melanocyte lineage revealed that both are required for proper hair pigmentation in a dose dependent manner (66). Furthermore, conditional ablation of the RBP-J gene in the melanocyte lineage led to defects in hair pigmentation followed by intensive hair graying (67). In line with this, administration of a GSI to wild-type adult mice resulted in gradual hair graying that was irreversible (68). Asides the Notch family members and the amyloid precursor protein, numerous other proteins can also be cleaved by γ-secretase, such as CD44, ErbB4, LRP, syndecan-3, p75 NTR, Apo ER2, DCC, Nectin-1α, E-cadherin and N-cadherin (69). For some of these proteins, such as CD44 binding to hyaluronic acid, the influence of γ-secretase could be to allow cells to become mobile by losing attachment to matrix components (70). For others, like ErbB4, γ-secretase converts it from a membrane-bound tyrosine kinase receptor to an intracellular protein with nuclear localization and the ability to bind and regulate multiple transcription factors (71). Interestingly, ErbB4 is for instance associated with the acquired resistance to ErbB2-inhibitors in HER2+ breast tumors, probably *via* a shift in the dependency towards ErbB4 signaling following continuous ErbB2 blocking, though additional resistance mechanisms should account (72). Regarding cadherins, γ-secretase activity may affect the epithelial-to-mesenchymal transition or the mesenchymal-to-epithelial transition, depending on the cadherin target [E-cadherin (73), or N-cadherin, respectively (74)]. However, the exact molecular mechanisms involved and the clinical implications remain to be elucidated. In conclusion, the fact that γ-secretase plays a broad biological role and cleaves multiple proteins make the use of inhibitors less attractive for Notch blockade.

#### Specific Notch-Targeting by Antibodies

**Figure 3:**
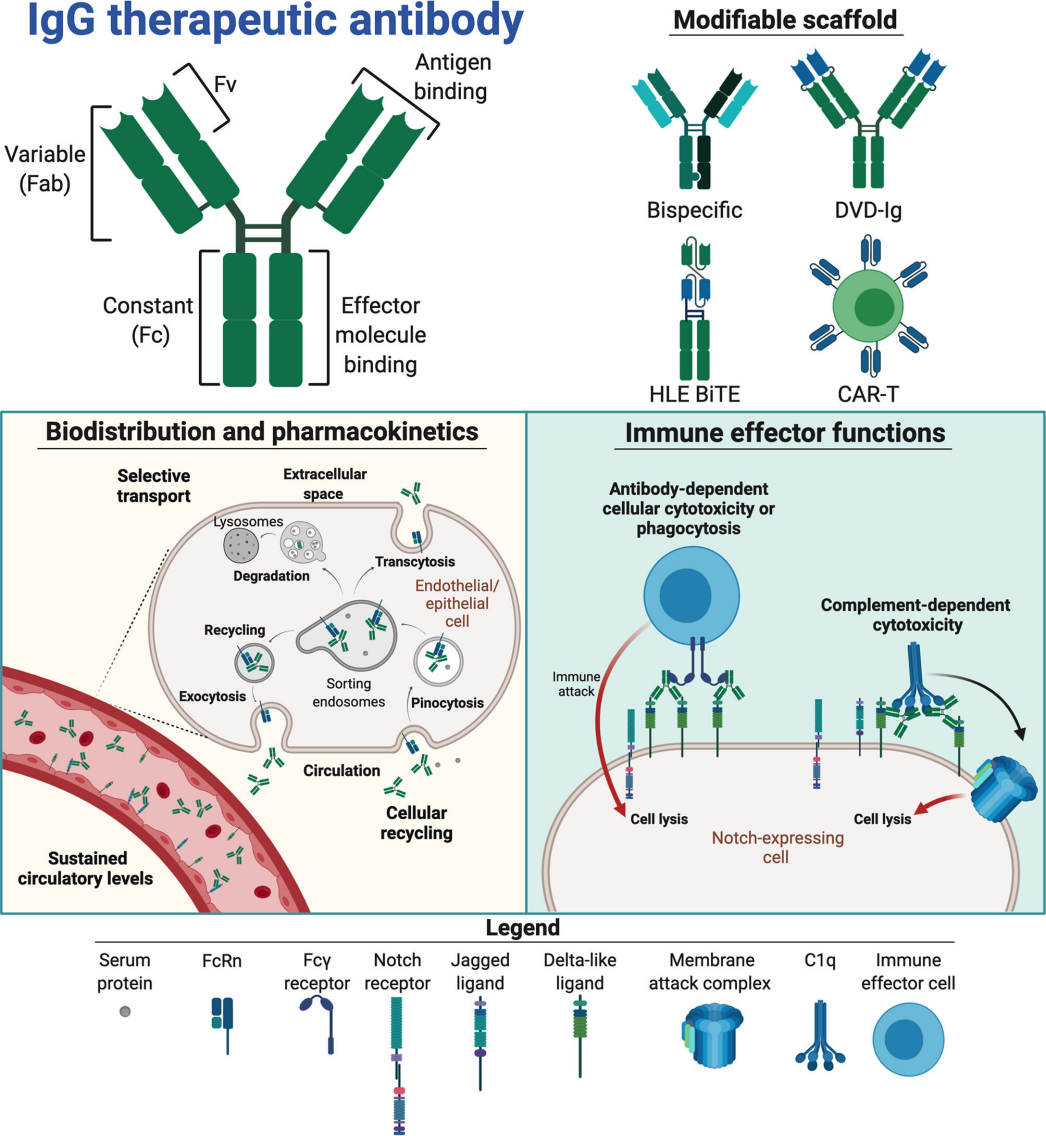
Therapeutic traits of mAbs for Notch interference. Notch interfering by mAbs is based on the therapeutic scaffold of immunoglobulin G (IgG, top left panel). The separate domains of IgG entail different capacities, linking antigen binding in the fragment variable (Fv) to the framework of fragment antigen binding (Fab) arms, which in turn are linked to the constant fragment crystallizable (Fc), containing effector molecule binding sites, *via* a disulphide-containing hinge. These domains constitute a modifiable scaffold and have been combined in various Notch-targeting moieties (top right panel) to create bispecific and T cell-based therapeutics. If incorporated into therapeutics, the IgG Fc facilitates binding to the neonatal Fc receptor (FcRn, bottom left panel), enabling cellular recycling and specific transcytosis. This may grant therapeutics a long circulatory half-life and affect their biodistribution. IgG Fc fragments may also engage Fcγ receptors and complement proteins (bottom right panel) to mark antigen-expressing cells for destruction *via* immune cells (antibody-dependent cellular cytotoxicity (ADCC) or phagocytosis (ADCP) or complement-mediated lysis (complement-dependent cytotoxicity, CDC). The figure was made in BioRender (https://biorender.com).

A more attractive and specific approach compared with pan-Notch inhibition, is the use of mAbs. The reason for this is that mAbs offer benefits over conventional pharmacotherapy in terms of potency, dosing frequency and specificity for the target. Also, mAbs are generally well tolerated, with off-target effects such as hypersensitivity reactions being relatively rare compared to target-dependent adverse events (75, 76). Thus, development of mAbs with tailored Notch-targeting properties may be a suitable approach. The most preferred molecular scaffold for design of mAbs is IgG1. The reason for this is that it has a long plasma half-life combined with the ability to induce potent effector functions (77). Structurally, it can be divided into a constant fragment crystallizable (Fc) part that mediates effector functions, which is linked to two fragment antigen binding (Fab) arms harboring target specificity (**Figure 3**, top left panel). This structure has several inherent traits that must be considered for Notch-targeting.

The long plasma half-life of three weeks at average is regulated by binding of the Fc part to the neonatal Fc receptor (FcRn) (78), which is a broadly expressed cellular receptor that rescues IgG from intracellular degradation *via* a recycling process (**Figure 3**, bottom left panel). In general, this provides favorably sustained systemic levels of therapeutic mAbs (78). Target availability, and whether the target is soluble or membrane-bound, does however affect the pharmacokinetic properties of mAbs. Regarding targeting of cell-bound Notch ligands and receptors, the pharmacokinetics will be affected through the so-called antigen-sink effect (79). While this will likely affect low-dosage efficacy, it may enable prolonged, specific Notch inhibition with appropriate dosing regimens (80).

FcRn also mediates transport of IgG across selective barriers such as the mucosal epithelium and the placenta (78, 81, 82). Transplacental transport may be particularly important to consider – due to the key developmental role of Notch signaling, Notch targeting may be harmful to the fetus. Notably, infant serum levels of the anti-TNFα mAbs infliximab and adalimumab may be equivalent to maternal levels following mAb treatment of rheumatoid arthritis (RA) during pregnancy (83, 84). This should thus be considered in treatment regimens and mAb design. One way to overcome this challenge will be to engineer the IgG:FcRn interaction by introducing amino acid substitutions in the Fc that attenuate receptor engagement, which will reduce both half-life and transcellular transport. Importantly, the FcRn binding site on IgG is distinct and distant from the binding site for classical Fcγ receptors (85). Another option is the use of the IgG2 subclass, which is less well transported to the fetus compared with the other three IgG subclasses. In this way, the transport properties of mAbs are customizable (78).

Furthermore, Fc-mediated effector functions may be initiated upon binding of mAbs to cell surface displayed targets and lead to target cell destruction by cross-binding to Fcγ receptors expressed by immune cells (antibody-dependent cellular cytotoxicity, ADCC and/or antibody-dependent cellular phagocytosis, ADCP) or engagement of the complement system (complement-dependent cytotoxicity, CDC) (**Figure 3**, bottom right panel). Such effector functions may be either instrumental to therapeutic efficacy, detrimental to Notch-expressing tissue or simply redundant, depending on both target and indication of the mAb. Under normal physiological conditions, these immune processes are tightly regulated by cells to prevent harmful immune activation (86–88). Thus, how novel therapeutics interplays with such regulatory mechanisms must be accounted for. For instance, in tumor targeting, potent effector functions are favorable, as this will enhance the ability of the mAb to induce tumor killing. However, if the aim is to block target-mediated function(s), or if effector functions are not required, they may even lead to adverse side effects. This potential risk may be minimized by the use of less effector potent subclasses, such as IgG2 or IgG4 (89). With the exception of the anti-DLL4 IgG1 mAbs MEDI0639 (90) and enoticumab (91), all anti-Notch mAb candidates under investigation to date are based on IgG2 (52, 92–94). Other options include hybrid mAb scaffolds, engineering of the IgG1 Fc to abolish effector molecule engagement or even excluding the Fc altogether by utilizing only Fab or the fragment variables (Fvs) (95–97). Furthermore, instead of the Fab arms, therapeutic proteins may be fused to the Fc to improve their pharmacokinetic profile and stability (98, 99). DLL4 has for example been fused to IgG1 Fc, creating a protein shown to suppress liver metastasis of small cell lung cancer both *in vitro* and *in vivo* (96). A mAb can also be engineered to harbor two Fab arms with different specificities, creating so-called bispecific mAbs. These can grasp onto two different targets, such as CD4/CD70 and HER2/EGFR (100–102). Such an approach may be attractive for Notch targeting to reduce on-target binding in off-target tissues and cells. The first bispecific Notch antibody nearing approval is the anti-vascular endothelial growth factor (VEGF)/DLL4 IgG2 navicixizumab, which in late 2019 was granted fast-track designation for FDA-approval in pretreated ovarian cancer (https://www.targetedonc.com/view/fda-fast-track-designation-granted-to-navicixizumab-for-heavily-pretreated-ovarian-cancer). Alternatively, Fvs from two different mAbs may be coupled together and fused to the Fc, thus creating dual-variable domain immunoglobulins (DVD-Igs). A DVD-Ig binding both DLL4 and VEGF is currently under clinical testing [ABT-165, (103)]. Importantly, this DVD-Ig harbors an IgG1 Fc containing two amino acid substitutions [L234A, L235A (LALA)] that reduce binding to effector molecules. Fv(s) can further be engineered to single-chain (Sc) Fvs capable of independent monovalent binding (104). ScFv as a building block has been utilized in design of anti-DLL3 chimeric antigen receptor T cells, creating an investigational anti-Notch cancer cell therapeutic [AMG 119, (105, 106)]. Similarly, anti-DLL3 has been used to direct CD3 expressing cytotoxic T cells to DLL3-expressing small cell lung cancer as a half-life extended bispecific T cell engager (HLE BiTE; AMG 757) that results in antitumor activity (107). The structure of AMG 757 consists of a bispecific ScFv fused in tandem to one arm of an IgG1 LALA Fc for half-life extension (107, 108). As such, a range of different antibody scaffolds with Notch targeting properties have reached human studies, resulting in a body of knowledge that may guide the design of future Notch targeting modalities.

#### Outcome of Target-Specific Notch Modulation

In animal studies, targeting of individual Notch receptors and/or ligands with mAbs exhibit reduced on-target intestinal toxicity compared to that seen following GSI treatment (109, 110). For example, use of a murine JAG1-targeting IgG1 mAb in a rat breast cancer xenograft model did not cause toxicity or any effects on body weight (111). Moreover, transient blockade of DLL1 and DLL4 with IgG1 mAbs in the peritransplant period provided durable protection against GVHD after allogeneic bone marrow transplantation without inducing limiting toxicity (109). However, mAb-based DLL-blockade in mice, rats or monkeys induced histopathological changes in the liver that were associated with development of subcutaneous neoplasms with uncertain malignant potential, which alludes to the fundamental role of the Notch system in mammalian physiology (112). On the other hand, blocking of DLL4 by the DVD-Ig ABT-165 was well tolerated in cynomolgous monkeys (103), leading to one completed and one currently recruiting clinical trial for metastatic colorectal cancer (NCT03368859) and various advanced solid tumors (NCT01946074), respectively. Furthermore, completed clinical trials of demcizumab have demonstrated DLL4 blockade to be associated with diarrhea, vomiting, fatigue peripheral edema and hypertension (113–115). These studies have enrolled patients with pancreatic cancer, ovarian-associated cancers and lung cancer, where the mAb has been combined with chemotherapy. One study of lung cancer also tackled demcizumab in combination with pembrolizumab, targeting the programmed cell death protein 1 (PD-1) (NCT02722954). These studies conclude on a tolerable toxicity profile at truncated dosing regimens (114), but further clinical testing of demcizumab is halted due to lack of sufficient therapeutic improvement (92). As reviewed elsewhere, other DLL4-specific mAbs (MEDI0639, enoticumab) have undergone similar trials and pause (92). Navicixizumab is however (as ABT-165) moving forward, having demonstrated notable antitumor activity in its first-in-human phase 1a trial of pretreated solid tumors (93).

Of note, targeting individual Notch receptors rather than activating ligands elicit side-effects similar to that of blockade of Notch activation through DLL4, with dose-limiting diarrhea being the most prominent adverse event during clinical testing of both the anti-Notch1 IgG2 brontictuzumab (NCT03031691) (116, 117), and the anti-Notch2/3 IgG2 tarextumab (118, 119). Importantly, both brontictuzumab and tarextumab were tolerable at adjusted dosages. The ability of mAbs to distinguish between Notch1 and -2/3 is likely essential in this context, as simultaneous inhibition of Notch1 and -2 results in intestinal toxicity (56, 110, 120). Still, neither brontictuzumab nor tarextumab is currently moving forward after failure to meet end goals in phase 1b and 2, respectively (https://www.globenewswire.com/news-release/2017/04/17/961251/0/en/OncoMed-s-Phase-2-Trial-of-Tarextumab-in-Small-Cell-Lung-Cancer-Does-Not-Meet-Endpoints.html).

Looking at alternative Notch ligands, DLL3 stands out as a target having reached clinical studies. In contrast to the previously discussed cell-surface displayed activating ligands, DLL3 resides predominantly in the Golgi apparatus and has fewer EGF-like repeats (121–123). DLL3 acts as a Notch antagonist and attenuates the effects of other DSL ligands (124), distinguishing it from the other Notch ligands. Therapeutic attempts aim to kill DLL3-expressing cells in neuroendocrine cancers, where DLL3 acts as an oncogenic driver, through either lymphocyte activation or antibody-drug conjugates (ADCs) (105, 121, 125). One ADC – rovalpituzumab tesirine – reached clinical trials, where side-effects included fatigue and peripheral edema before it failed to meet its end goal (https://news.abbvie.com/news/press-releases/abbvie-discontinues-rovalpituzumab-tesirine-rova-t-research-and-development-program.htm).

Taken together, evidence suggests that 1) blockade of individual Notch ligands and/or receptors is attainable without intolerable adverse effects and 2) efficacy of targeting Notch ligands, in particular DLL4, is dependent on both indication and combinatory treatment strategy. Importantly, in-human testing of anti-Notch mAbs have yet to reveal any disastrous side-effects, such as those seen in the 2006 study of the anti-CD28 mAb TGN1412 (126). While the clinical efforts on anti-Notch mAbs so far have centered on various, often difficult to treat cancers, the growing body of knowledge surrounding specific Notch blockade may pave the way for development against other immuno-dysregulations, such as inflammatory diseases. Such attempts must be tailored to gain specific therapeutic benefits with limited adverse and toxic effects. In addition, the failure to meet the end goals of the cancer trials, might also be related to the supporting role of Notch in maintaining a pro-inflammatory phenotype of various immune cell subsets (**Table 1**), which is also required for eliciting an anti-tumor immune response. As such, targeting Notch in other pathophysiologies, where inflammation is the driving force for the disease manifestations, may prove a more valid approach.

#### Targeting the Notch System in Combination With Other Therapeutic Regimens

In view of targeting Notch signaling in inflammation and the increasing emphasis on combination therapy for chronic inflammatory diseases and cancer, a few recent reports are noteworthy. A significantly improved outcome in a mouse xenograft model of glucocorticoid-resistant T cell acute lymphoblastic leukemia (T-ALL) was reported (127), using a combination of the GSI, dibenzazepine, with the glucocorticoid, dexamethasone. Dexamethasone was shown to counteract the lethal gut toxicity induced by the GSI, and the combination therapy induced apoptosis in xenografts of T-ALL cell lines in mice, to a greater extent than either dexamethasone or dibenzazepine alone. Corroborating with these findings, Samon and colleagues (128), reported a synergistic antileukemic response between the clinically relevant GSI, PF-03084014, and dexamethasone in human T-ALL cell lines, primary patient samples, and in a xenograft model of glucocorticoid-resistant T-ALL. Mechanistically, the GSI increased the transcriptional up-regulation of the glucocorticoid receptor and its target genes, suggesting increased cytotoxicity due to synergistic increase in the glucocorticoid activity. Moreover, gastro-intestinal toxicity induced by PF-03084014 was reversed by dexamethasone, although the precise mechanism is not clear. Notably, the therapeutic efficacy of brontictuzumab against Notch1 was also significantly enhanced in combination with dexamethasone in T-ALL xenografts (129), implying that the effects are not dependent on pan-Notch inhibition or the pharmacodynamics of the GSI but rather result from synergy between the Notch1 inhibition and the increased glucocorticoid receptor activity. This approach, if it can be translated to human patients, might have benefits not only over current treatment of T-ALL, but also for the treatment of chronic inflammatory diseases, where Notch signaling is involved in disease pathogenesis. Glucocorticoids are often used in the clinic to treat or prevent inflammation either alone or in combinational therapies. However, a wide range of dose-related and duration of treatment-related adverse effects of glucocorticoids including osteopenia, skin atrophy, diabetes mellitus, hypertension, muscle atrophy, and cataract formation, have been reported (130).

Moreover, almost 1/3 of patients with ulcerative colitis, asthma, systemic lupus erythematosus, RA, and uveitis, have a disease that is refractory to glucocorticoid therapy (131). Since a synergistic benefit is most likely to occur if therapeutic agents affect different, yet complementary pathways in disease pathogenesis, combinational treatment of Notch inhibitors along with anti-inflammatory therapeutics could be an alternative in chronic inflammatory diseases, refractory to other treatment regimens.

Regarding synergy, it must be noted that the closest to clinical success of mAbs in a Notch context to date is when combining DLL4 and VEGF blockade. This is underlined by observations in preclinical studies of enoticumab combined with the anti-VEGF IgG1 Fc-fusion (aflibercept) in different cancer models (132, 133), ABT-165 in mouse xenografts (103) and in clinical studies of navicixizumab (93). Two additional anti-VEGF/DLL4 bispecific IgG1 antibodies exist and have shown promising results in preclinical cancer models – HB105 and HB-32 (134, 135). Of note, chemotherapy is standard of care for most indications mAbs are investigated for in current clinical trials. Thus, the mAbs are administrated in combination with various chemotherapeutic agents, such as paclitaxel for navicixizumab in ovarian, peritoneal and fallopian tube cancer (NCT03030287). The rationale for this is not new; DLL4, like VEGF, is targeted to disrupt tumor angiogenesis, with an intent to prevent further tumor growth and/or improve delivery of alternative therapeutics through normalized tumor vasculature (136–139). Notably, DLL4 blockade may both enhance chemotherapeutic efficacy and elicit tumoricidal effects in anti-VEGF-resistant tumors (137, 138). Thus, Notch-targeting therapeutics may function as both replacement options and as catalysts for enhancing efficacy of existing therapies in difficult-to-treat cases.

### Notch Signaling in Chronic Inflammatory Diseases

There are several reports of dysregulated Notch signaling in clinical samples from patients with chronic inflammatory diseases. For example, transcription levels of NOTCH1 and HES1 were significantly elevated in colonic mucosal biopsies from patients with ulcerative colitis (140). By analyzing gene expression in tissue from arteries of patients with giant cell arteritis and healthy individuals, Wey and colleagues found abundant expression of JAG1 and NOTCH1 in vasculitic arteries (141). Jagged1 protein was expressed on endothelial cells from patients with giant cell arteritis, but not on endothelial cells from healthy individuals. Moreover, Notch1 protein was up-regulated on circulating CD4+ T cells in patients with giant cell arteritis compared to healthy controls. Likewise, circulating CD4+ T cells and Th2 cells in patients with asthma have been found to have higher levels of NOTCH1 and NOTCH2 (142). Higher levels of active NOTCH1 have also been observed in endothelial cells of inflamed human appendix (143). Such clinical observations, but also experimental studies involving different animal models of inflammation, support a pro-inflammatory role of Notch. In the following sections, we discuss some chronic inflammatory diseases where Notch signaling seem to play a role in pathogenesis, and thus, may represent a potential novel therapeutic target.

#### Giant Cell Arteritis

Giant cell arteritis is a chronic vasculitis of large- and medium-sized vessels. Although it is a systemic illness and vascular involvement may be widespread, symptomatic blood vessel inflammation most frequently involves the cranial branches of arteries originating from the aortic arch. Visual loss is the most feared complication, resulting from vascular stenosis and occlusion of the central retinal artery (144). A critical step in the disease process is the activation of pro-inflammatory T cells in the vessel wall. It has been demonstrated that treatment with a GSI or soluble Jagged1 to inhibit Notch-dependent signaling, effectively suppressed vascular inflammation in a humanized mouse model, in which human T cells form inflammatory lesions in human vessel were used as xenografts (145). Notch blockade suppressed activity of IFN-γ-producing Th1 cells and made IL-17-producing Th17 cells almost undetectable. This effect may have resulted from a disrupted communication between Notch1-expressing, circulating CD4+ T cells and vascular smooth muscle cells or endothelial cells expressing Jagged1 (141). The latter was shown to up-regulate expression of Jagged1 in response to excess circulating VEGF, thereby educating circulating CD4+ T cells and inducing T cell activation. Not only that, aberrant Notch4 signaling was recently identified as a cause of defective CD8+ Treg cell function, promoting vascular inflammation *via* accumulation of endosomes and sequestering of NADPH oxidase 2 intracellularly (146). Together, these studies point to the immunostimulatory functions of endothelial Jagged1 and the importance of normal Notch4 signaling in anti-inflammatory CD8+ Treg cells, emphasizing the therapeutic potential of targeting Notch in vasculitis.

#### Rheumatoid Arthritis

RA is an autoimmune disease that affects approximately 1% of the worldwide population (147). The etiology is not fully understood, but involves both genetic and environmental factors. The main autoimmune target in RA is the synovium, facilitating the development of a new synovium with aberrant qualities. This transformed, thickened synovium is called pannus, and harbors invading properties capable of eroding cartilage and bone, thus eventually destroying the functional joint. A leading pannus front involving pathologically activated osteoclasts is fueled by microenvironmental cytokine production, angiogenic vessel reorganization and endothelial activation responsible for leukocyte recruitment, including Th1, Th17, monocytes and neutrophils (148).

Several studies in murine models of arthritis have focused on the *in vivo* anti-inflammatory potential of Notch inhibition. Most of these have employed either GSIs or Notch1-inhibition, though a growing body of selective modulations involving other members of the pathway is reported (149–151).

The impressive anti-inflammatory effect of GSIs has been demonstrated also in these arthritis models; in both collagen induced arthritis (CIA) (152–154) and collagen antibody-induced arthritis (CAIA) (153), as well as in transgenic TNF-overexpressing (TNF-Tg) mice (155). As a strategy to circumvent the intestinal on-target side effects of global Notch-inhibition, nano-vesicle delivery systems have been employed (156); and a similar strategy showed promise with Notch1-siRNA either alone (157) (in CIA mice) or in combination with the disease modifying anti-rheumatic drug methotrexate (158) (in rats). Genetically engineered Notch1 Antisense-expressing (NAS) mice, where global expression of Notch1 is reduced, are less arthritogenic than controls (152, 153), while conditional myeloid overexpression of NICD1 trended towards worsened arthritis in one study (159). Antibody-mediated inhibition of Notch1 signaling by an IgG1 anti-human and anti-mouse Notch1 led to a modest attenuation in arthritis severity and paw swelling (151).

Other Notch member-specific modulations have successfully attenuated experimental arthritis using HMD1-5 hamster anti-mouse Dll1 IgG (150, 160) and anti-human and anti-mouse Notch3 IgG2a (151) mAbs. On the other side, anti-Jagged1 mAb-treated mice *worsened* CIA in one study (149), further corroborating the importance of specific targeting selective members of the Notch axis.

How Notch drives the arthritogenic inflammation is thus multifaceted, but involve inflammatory events like endothelial activation and leukocyte recruitment [e.g (143).,], leukocyte activation and function (discussed above), and pathologic angiogenesis (see AMD-section below). Notch1-signaling is involved in hypoxia-induced angiogenesis and potentially also in VEGF/Ang2-induced expression of IL6, IL8, MMP-2 and MMP-9 (161). On the other hand, Notch1 seems to inhibit osteoclastogenesis, while DLL1/Notch2-signaling promotes osteoclast formation and bone resorption (150). Endothelial DLL1 is also involved in Notch2-mediated differentiation of the Ly6C^low^ monocyte subtype (162), a subtype involved in both initiation and progression of experimental arthritis (163). Notch also has important functions in fibroblast-like synoviocyte, because DLL1, expressed on synovial macrophages, has the potential to activate Notch2 (upregulated on synoviocytes by TNFα), to secrete pro-inflammatory cytokines and matrix metalloproteinases (164). In addition, Notch3 is involved in the differentiation and arthrogenic expansion of a special subset of CD90-positive fibroblasts in the inflamed synovium (151).

A more detailed understanding of the various Notch members’ contributions to RA pathogenesis may aid future treatment strategies, but requires enhanced focus on possible on-target side effects to enable identification of the truly beneficial targets and/or strategies.

#### Systemic Lupus Erythematosus

Systemic lupus erythematosus (SLE) is a systemic autoimmune disease affecting different organ systems due to the deposition of immune complexes that activate complement (165). Disturbed apoptosis, following UV radiation and impaired removal of apoptotic material, may lead to an increased burden of nuclear antigens, and development of anti-nuclear auto-antibodies (ANA), such as, anti-double stranded DNA (anti-dsDNA), anti-histone, and anti-Sm [reviewed in (166)].

Notch signaling may affect SLE in several ways. GSI treatment reduced elevated numbers of double negative (CD4-/CD8-) T cells and several other disease parameters in the well-studied murine MRL/lpr model of SLE (167). Focusing on the putative role of the different macrophage activation subtypes in SLE pathogenesis [reviewed in (168)], GSI treatment may also ameliorate lupus by impeding the polarization of a particular macrophage subtype (characterized among others by the markers MHCII, CD86, TNFα, IL-10^high^, IL-12^low^) in a murine SLE model, generated by immunization with activated lymphocyte-derived DNA (169).

Moreover, cleaved Notch1, cleaved Notch2, and Jagged1 have been shown to be expressed on podocytes in proteinuric nephropathies including lupus nephritis, one of the most serious manifestations of SLE, and their level of expression correlated with the amount of proteinuria, across all disease groups (170). Stronger mechanistic data, obtained by genetic deletion or knockdown technology, revealed that Notch3 is involved in the progression of nephritis, by promoting migratory and pro-inflammatory pathways (171). In line with these findings, persistent Notch activation induced podocyte death, and it has been suggested that Notch acts as a regulator of the balance between podocyte death and regeneration during glomerular disorders (172).

Although the above-mentioned studies demonstrate efficacy of Notch inhibition in experimental SLE or nephritis models, and suggest that Notch inhibition could be an attractive new therapeutic approach, it should be mentioned that clinical observations appear to point in other directions. Comparing T cells from SLE patients with those from healthy controls, the former failed to up-regulate Notch1 expression upon *in vitro* stimulation. They also exhibited significantly less up-regulation of Notch 1 expression compared with SLE patients with remission (173), and therefore, a reversed correlation with the disease severity. Reduced Notch1 levels in human T cells from SLE patients are also associated with increased IL-17A production (174). However, while GSI treatment inhibits Notch signaling in all receptors and all cell types, signaling *via* Notch1 in one cell type could possibly elicit responses that counteract the global Notch effect.

#### Multiple Sclerosis

Multiple sclerosis (MS) is a chronic inflammatory disease of the central nervous system (CNS) characterized by an auto-reactive, presumably Th1/Th17-initiated, immune response targeting myelin. The disease most frequently follows a relapsing-remitting course, eventually leading to chronic progression and disability as irreversible damage to axons become prominent (175). Axonal damage is preceded by a loss of oligodendrocyte maturation and their remyelinating capacity.

Notch signaling prevents the neural progenitor stem-cell pool from developing into neurons, thereby ensuring oligodendroglial precursor cell (OPC) potential (176). However, up-regulation of Jagged1 on activated astrocytes inhibits oligodendroglial maturation through a canonical Notch1-Hes5 dependent response in OPCs (177, 178). Thus, since OPCs are readily present but fail to mature in MS patients, it has been suggested that therapeutic inhibition of Notch signaling could favor remyelination in MS lesions (179). However, other studies propose that Notch signaling do not inhibit remyelination (180, 181), but rather promotes it (182, 183). It is possible that Notch signaling serves to maintain the OPC pool (their proliferation and migratory potential), while also providing local, ligand and/or receptor specific environmental cues from demyelinated axons to modulate late stages of oligodendrocyte maturation.

Regardless of the underlying mechanisms, the overall effect of Notch inhibition in neuro-inflammation appears beneficial in several studies, where various approaches have been used. In the experimental autoimmune encephalomyelitis (EAE) model in mice, GSIs reduce clinical and histopathological signs when given either systemically or intraventricularly (30, 184).

While GSIs are capable of penetrating the blood-brain barrier and mount a direct effect on remyelination, mAb therapies given systemically probably exert their effects through modulating the peripheral immune response, and only indirectly affect the CNS. Nevertheless, anti-DLL1 (HMD1-5 hamster anti-mouse Dll1 IgG) (185, 186) or anti-DLL4 (HMD4-2 hamster anti-mouse Dll4 IgG) (10, 187) given systemically reduced the disease severity in mouse models. By contrast, anti-Jagged1 treatment worsened the disease, apparently through inhibiting a Jagged1-dependent up-regulation of splenic IL10-producing cells (185). Altogether, there seem to be a relative consensus that inhibitions of the delta-like ligands cause a reduction in the Th1- and Th17- responses and their CNS infiltration and/or accumulation, perhaps by activating Notch3 (184). The release of Treg-inhibition is suggested as an additional means of action during DLL4 inhibition (10). Some of the positive preclinical findings observed in MS animal models with GSIs might also be the result of Notch inhibition in CNS-resident microglia (188), or tissue infiltrating monocytes/macrophages (189). Reduced Notch2-expression on ‘classical’ (CD14+) monocytes has proved its potential as biomarker to predict drug-neutralizing antibody development in IFNβ-naive MS patients, and a functional link between Notch2-activity and the differentiation into ‘non-classical’ (CD14^low^CD16+), pro-inflammatory monocytes was indicated and certainly advocate future studies (190).

Given the possibility that Notch signaling affects both the inflammation and the failed remyelination occurring during MS pathogenesis, future strategies could focus on enhancing CNS delivery of specific Notch modulators administered systemically (191). Targeting specific ligands and receptors could ensure the beneficial effects of Notch inhibition in inflammation and, putatively, in remyelinisation.

#### Systemic Sclerosis

Systemic sclerosis (SSc) is a chronic fibrotic disease of unknown etiology that affects the skin, and several internal organs (192). Histopathological hallmarks of early stages are perivascular inflammatory infiltrates and a reduced capillary density (193). Later stages are characterized by an excessive accumulation of extracellular matrix (ECM), caused by persistently activated fibroblasts (192). The resulting fibrosis disrupts the physiological structure of the affected tissues, interferes with proper organ function and is the major cause of death in SSc patients (192, 193). Some advances have been made to treat vascular complications, but no treatment has shown convincing effectiveness to reduce skin and visceral fibrosis. Thus, SSc is still considered incurable.

While research has long been focusing on pro-fibrotic cytokines such as TGFβ, PDGF, MCP-1, IL-4, IL-13 and endothelin, accumulating evidence highlights key roles of morphogen pathways like Wnt, Hedgehog and Notch in the pathogenesis of SSc and other fibrotic diseases (193).

An increasing amount of evidence suggests that the Notch pathway is implicated in the fibrosis development that characterizes SSc. Indeed, Notch1 was activated in the lesional skin of SSc patients and in their fibroblasts (194, 195). Mice with ROS-induced SSc, bleomycin-induced SSc and Tsk1-mice also displayed elevated levels of NICD, overexpression of the ligand Jagged-1, and increased transcription of the target gene HES-1 in the skin and lungs. This accumulation of NICD was associated with the over activation of ADAM17 (also known as tumor necrosis factor-α-converting enzyme, TACE) (194). Moreover, treating mice with a GSI could reduce the collagen content in both skin and lungs and the production of autoantibodies, thus preventing the development of SSc in various *in vivo* models (192, 194, 195). Similarly, treating SSc-mice with Notch siRNA prevented dermal thickening and fibrosis (192). In the same study, inhibition of Notch signaling prevented the development, of dermal bleomycin-induced fibrosis and inflammation-independent fibrosis in TSK-1 mice. These data suggest that targeting of Notch directly reduces collagen synthesis in fibroblasts and might be effective in the early inflammatory stages of SSc, as well as, in later stages when the inflammatory infiltrates have been resolved. These results are also consistent with those of a study in which the GSI DAPT prevented hypochlorite-induced fibrosis (196).

In summary, the Notch pathway is activated in SSc and inhibition of Notch signaling seems to exert potent anti-fibrotic effects in preclinical models. Thus, the Notch signaling pathway might be a promising molecular target for anti-fibrotic therapeutic approaches, however, the relevant evidence from clinical trials is yet lacking.

#### Inflammatory Bowel Disease

Ulcerative colitis (UC) and Crohn’s disease (CD) are the two major phenotypes of inflammatory bowel disease (IBD). Like many other chronic inflammatory diseases, IBD is thought to occur in genetically susceptible individuals exposed to environmental risk factors. Notch signaling plays an indispensable role in gut formation, from the earliest stages of primitive gastrointestinal tube development, to the differentiation and proliferation of both epithelial stem cells and mature cells (197). Beyond its critical roles in normal development and homeostasis of the gut, Notch signaling may also be crucial to the maintenance of the intestinal architecture and barrier function (198).

Functional studies of the role of Notch in the gut have for the most part used either GSIs or genetic modulation of RBP-J. GSIs cause a dose-dependent conversion of enterocyte progenitors to the secretory cell fate, accompanied by activation of the basic helix-loop-helix transcription factor Atoh1/Math1, and down regulation of Hes1 in intestinal crypt progenitors (56). A similar phenotype is obtained by knocking out RBP-J in the intestinal epithelium. Most of the effects observed are probably the result of Notch1 inhibition, since blocking Notch2 with a mAb (anti-human and anti-mouse IgG1, YW169.60.79) did not induce any noticeable effect in the intestinal morphology (110).

It has been demonstrated that Notch signaling increases IL-6 production through an NFκB and RBP-Jκ-mediated pathway in colon epithelial cells, and also that, transient blockage of Notch signaling reduces intestinal inflammation during experimental colitis (199). On the contrary, mice harboring intestinal epithelial cell-specific deletion of RBP-J spontaneously developed chronic colitis characterized by the accumulation of Th17 cells in colonic lamina propria (200). Moreover, absence of colonic Notch1 increased disease severity in acute dextran sulfate sodium (DSS)-colitis (197). Although supporting evidence of a pro-inflammatory role of Notch in experimental IBD exists (199), it’s probably unlikely that blocking the Notch signaling, at least with pan-Notch inhibitors or Notch1-selective inhibitors, is a promising future strategy for treatment of intestinal inflammation. Convincing evidence now indicates that Notch signaling ensures integrity and homeostasis of the intestinal epithelium, and thus any possible positive effects of Notch inhibition in colitis may be overshadowed by the unwanted effects. It should however be emphasized again that individual Notch ligands and receptors can have divergent functions, and future functional studies in IBD models should examine the effect of individual ligand and/or receptor blockade.

#### Allergic Asthma

Asthma is characterized by persistent airway inflammation, airway hyper responsiveness and mucin hypersecretion. The pathogenesis is complex and involves both genetic and environmental factors (201). Allergen-specific IgE is elevated and the Th2 cytokines IL-4, IL-5, IL-9, and IL-13 are secreted by T helper cells followed by subsequent activation of mast cells, infiltration of eosinophils and airway smooth muscle constriction (202). After antigenic stimulation, naive CD4+ T cells differentiate into two distinct helper T cell subsets; Th1 and Th2 cells (203). It has been generally accepted that disturbed T helper cell balance plays an important role in the pathogenesis of asthma (204).

In a mouse model of asthma, siRNA-mediated Notch1 blockade decreased IL-4 and increased IFN-γ in activated lung T cells (205), suggesting that Notch1 could play a role in regulating the Th1/Th2 imbalance (205, 206). Other studies have also reported that pharmacologic inhibitors of Notch signaling can reduce allergic pulmonary inflammation by modulating Th1 and/or Th2 responses (203, 207, 208). These observations may be mechanistically explained by a critical requirement for Notch to license the Th2 response *via* promoting lymph node egress of effector Th2 cells (209). Moreover IL-17-producing T helper cells (Th17) are important players in asthma pathogenesis (203). Numerous studies have shown an increase of Th17 cells in the inflammatory airway (205). Therefore, suppressing Th17 response may serve as a promising therapeutic strategy (210). An important role for Notch signaling in Th17 cell differentiation was recently emphasized since, GSI treatment of mice with ovalbumin-induced asthma, ameliorated disease development (211). This treatment also suppressed the Th17-cell responses in spleen and reduced the IL-17 levels in serum. It is worth noting that circulating T cells from asthma patients exhibit increased Notch expression (142). Thus, targeting Notch signaling may represent a promising therapeutic strategy to treat allergic asthma.

#### Uveitis

Uveitis is inflammation of the uvea, which is made up of the iris, the ciliary body and the choroid. It may be caused by infections, eye traumas, toxins that penetrate the eye, or autoimmunity. It is usually an isolated illness but can also be part of a systemic condition, such as ankylosing spondylitis, juvenile rheumatoid arthritis or Behcet’s disease. Autoimmune uveitis is thought to be related to an aberrant T cell-mediated immune response. Notably, increased activation of the Notch pathway has been demonstrated in CD4+ T cells from patients with active uveitis (212). Moreover, it has been shown that DLL4-induced signaling is critical for the development of experimental autoimmune uveitis (EAU) in mice (213), and that global inhibition of Notch signaling with DAPT, reduced the levels of various proinflammatory cytokines (214). In this model, auto-aggressive CD4+ T cells are the major pathogenic population. Anti-DLL4 mAb (HMD4-2 hamster anti-mouse Dll4 IgG) given systemically during the induction and effector phases reduced the severity of EAU (213). GSI treatment in mice has also been shown to suppress EAU progression, presumably *via* decreased IL-22 production in CD4+ T cells. This effect was even observed when treatment was administered 13 days after immunization (215). Furthermore, the role of Notch might be more nuanced than captured by these studies, because Notch1 knockdown in infiltrating Tregs contributed to the suppression of EAU (216). Together, these findings indicate that Dll4-mediated Notch signaling is critical for EAU development during the induction phase, but also that inhibition of Notch1 is important for the Treg-mediated suppression of EAU. Perhaps selective Notch blockade could hold promising therapeutic potentials in autoimmune uveitis. Future studies are warranted to explore this possibility.

#### Age-Related Macular Degeneration

A concrete body of evidence suggests that low-grade chronic inflammation contributes to the progression of human diseases that were previously not considered as inflammatory disorders, including age-related macular degeneration (AMD) [reviewed in (217)]. In early “dry” AMD, extracellular deposits, known as drusen, accumulate between the retinal pigment epithelium (RPE) and Bruch’s membrane. Most likely, chronic inflammation and complement activation play some roles in the formation of these, and drusen material itself is pro-inflammatory (218).

Although the relationship between drusen formation and AMD is only correlative, increasing accumulation of the former enhances the risk of developing advanced disease; geographic atrophy or exudative (wet) AMD. Wet AMD is characterized by invasion of abnormal choroidal blood vessels (choroidal neovascularization, CNV), and fluid leakage into the retina. At least for wet AMD, be it in AMD patients or experimental CNV, evidence supports the involvement of immune cells (219), such as T cells, macrophages and monocytes that have been identified in eyes from AMD patients (220, 221).

To date, the role of Notch signaling in AMD pathogenesis remains elusive and warrants further research. As previously mentioned, the complement pathway is involved in drusen formation and emerging evidence suggests its causative role in the development of AMD (222). Interestingly, newly identified functions of complement include a role as a nexus for interactions with other effector systems, including the Notch signaling (223). The complement regulator CD46 is a potent co-stimulator for the induction of IFN-γ-secreting Th1 cells and their subsequent switch into regulatory T cells. Jagged1 was recently identified as a new physiological ligand for CD46, and CD46 activation on T cells was shown to regulate the expression of Notch and Notch ligands (224). It is thus legitimate to hypothesize that dysregulation of Jagged1 and/or CD46, leading to Notch hyperactivity, would increase Th1 responses that in turn may contribute to low-grade inflammation in AMD (225). Consequently, interference with Notch signaling could have beneficial effects in AMD progression.

Undoubtedly, Notch plays an important role during pathological angiogenesis, and pan-Notch inhibition was shown to increase the CNV volume, in a study of laser-induced CNV in rats (226). Many of the changes observed were presumably due to the modulation of the endothelial Notch signaling, as mice with conditionally deleted RBP-J in endothelial cells, display patterns similar to those observed after pan-notch inhibition, such as increased leakage shown on angiography and larger CNVs after laser photocoagulation compared to control mice (227). In contrast to treatment with a pan-Notch inhibitor, intravitreal injections of a Jagged1 peptide reduced the CNV volume (226). Nevertheless, Jagged1 is a potent proangiogenic regulator (228), and thus, it is unclear whether the effects observed were due to inhibition of Jagged1-Notch, cell-to-cell interactions or activation of Notch. Administration of Jagged1 peptide reduced sprouting in the postnatal mouse retina (228), suggesting an inhibitory effect. It is tempting to speculate that some of the effects on laser-induced CNV lesions after Jagged1 modulation could be the result of immune cell suppression. Interestingly, a recent genome-wide association study (GWAS), showed an association between AMD and single-nucleotide polymorphisms of the NOTCH4 gene (229). Most of the association signal came from CNV, but the subgroup analysis also suggested an association with geographic atrophy. Thus, Notch signaling, such as its role in the function of immune cells, may be of particular importance.

Notch also has a role in mediating the cellular response to hypoxia through crosstalk with hypoxia-inducible factors 1- and 2-α (HIF1α, HIF2α) (230, 231). Inhibiting Notch signaling has been suggested as a therapeutic approach to prevent hypoxia-induced tumor invasion in uveal melanoma (232). In the same study, hypoxia was found to increase the levels of Notch ligands, including Jagged1. Blocking Notch signaling could thus be a promising approach to reduce hypoxia-mediated stress and thereby pro-angiogenic signaling, both suggested as driving factors in wet AMD (233). A recent study of combined targeting of HIF1α and VEGF in a hypoxia-driven model of retinal neovascularization concluded on beneficial effects of combinatory treatment (234). This raises the hypothesis that the interplay with the hypoxic pathways may also enable Notch blockade to increase the efficacy of the current VEGF-based treatment regimens in neovascular eye diseases. Hypoxia has also been found to up-regulate Notch3 in lung tissue, alluding to the hypothesis of Notch3 serving as a potential therapeutic target to reduce pulmonary arterial hypertension (235). Interestingly, Notch3 has also been shown to be crucial in pathological neovascularization in an oxygen-induced retinopathic model, by stimulating production of angiopoietin-2 and thereby driving angiogenesis (236). Angiopoietin-2 is a known disease driver in AMD and is being targeted by the bispecific mAb IgG1 faricimab, currently in clinical trials for both diabetic macular edema (NCT04432831, phase 3) (237) and wet AMD (NCT03823287, phase 3) (238). Targeting Notch3, to inhibit angiopietin-2 production, could thus be a potential therapeutic avenue for mAb-based anti-Notch approaches that could act upstream of strategies currently in use and/or development.

In addition to their potential effects in eye diseases, Notch-interfering compounds may have an important niche for ocular delivery as the molecular format can be engineered to minimize systemic exposure. This is an active field in mAb design, which has not yet arrived at a clear conclusion. Important perspectives are tissue penetration into the retina, active and passive transport out of the ocular space, interaction with effector molecules and systemic clearance rate (239). Following injection into the eye, therapeutics eventually end up in the circulation, where the aforementioned inherent traits of mAbs may allow them to persist for prolonged periods of time. Therefore, it is not given that anti-Notch mAbs previously tested in clinical trials, would be optimal in an ophthalmological setting. Thus, both the optimal disease-specific Notch target(s) and the molecular format(s) remain to be determined.

#### Atherosclerosis

Atherosclerosis is an inflammatory disease and not merely the passive accumulation of lipids within artery walls (240). The chronic inflammatory process involving the arterial endothelium that results in the complications of atherosclerosis may be caused by a response to the oxidative components of the modified low-density lipoprotein (LDL), chronic infection, free radicals or other factors (241). Recent evidence has shown that Notch signaling was activated in luminal endothelial cells at atherosclerotic lesions from human aortas (242). Enforced Notch activation also resulted in endothelial cell senescence and upregulated expression of several molecules implicated in the inflammatory response. Additional experimental data, point to the potential of targeting the Notch signaling in atherosclerosis (243). Blockade of DLL4-Notch signaling using an anti-DLL4 mAb (HMD4-2 hamster anti-mouse Dll4 IgG), attenuated the development of atherosclerosis, diminished plaque calcification, improved insulin resistance, and decreased fat accumulation in LDL-receptor-deficient mice fed with a high-fat, high-cholesterol, diet (244). In the same study, DLL4 inhibition appeared to reverse the NF-κB/MCP-1-mediated macrophage accumulation in arteries and adipose tissues. Furthermore, systemic administration of a GSI, reduced plaque areas and fatty streak content in the aortic sinus of apolipoprotein E-deficient mice, fed with a Western diet (245). Similarly, this treatment also suppressed the migratory activity of primary murine peritoneal macrophages and of a macrophage cell line (RAW 264.7) and reduced the expression of ICAM-1, resulting in significantly decreased macrophage infiltration in the atherosclerotic plaques in mice (245). As previously discussed, activation of Notch probably promotes atherosclerosis by inducing a pro-inflammatory activation phenotype in macrophages at the expense of the anti-inflammatory subtype. Even though the Notch signaling has attractive potentials both as a biomarker and as a therapeutic target candidate in atherosclerosis (246), more studies are needed to dissect the precise roles of the various Notch members in the atheromatous plague formation and to explore their individual potentials as novel treatment strategies.

#### Graft-Versus-Host Disease

The GVHD induced by donor-derived T cells remains the major limitation of allogeneic bone marrow transplantation (allo-BMT). Current strategies to control GVHD rely on global immunosuppression, however, these strategies do not work in all cases and also decrease the anticancer activity of the allogeneic graft. A critical role for the Notch signaling was discovered in pathogenic host-reactive T cells after allo-BMT (247). A dominant negative Mastermind-like (DNMAML) pan-Notch inhibitor was conditionally expressed in mature CD4+ and CD8+ T cells, leading to reduced GVHD severity. Furthermore, Treg cell-specific deletion of Notch signaling components, protected against full MHC-mismatched GVHD (64). A recent report has provided additional evidence underpinning a clinical relevance (109); transient DLL1 [anti-human and anti-mouse Dll1 IgG1 (109),] and DLL4 [anti-human and anti-mouse Dll4 IgG1, YW152F (138),] inhibition using specific humanized mAbs was sufficient to provide long-lasting protection against GVHD in mice models. Protection was associated with persistent Treg expansion. Notch1 inhibition also controlled GVHD, but led to treatment-limiting toxicity. These findings highlight the therapeutic potential of targeting individual Notch receptors or ligands as a new strategy for controlling GVHD after allo-BMT.

## Conclusions and Future Perspectives

To date, no compounds interfering with the Notch signaling are approved for patients with inflammatory diseases, neither have any reached clinical trials investigating the potential thereof. Clinical studies inhibiting the Notch system have so far focused on cancer, although an increasing interest in defining the roles of Notch in inflammation is clearly evident. Several pharmaceutical and biotech companies have designed pan-Notch inhibitors, Notch receptor or ligand subtype-specific mAbs, and an extensive overview of ongoing and completed clinical studies stratifying Notch targeting strategies in patients with cancer have been reported (52). Based on the available preclinical data from mouse models of inflammation, we propose that modulating the Notch signaling could be an attractive new therapeutic option in several diseases where inflammation plays a fundamental role. Nevertheless, translating the preclinical findings from these models into the clinic remains a challenge. **Table 2** summarizes the antibodies used in animal experimentation, up to date. The ubiquitous expression of Notch receptors and ligands on many cell types makes intolerable side effects a distinct possibility, and poses challenges on both therapeutic mode of action and delivery. In these ways, Notch can indeed be said to be a “high hanging fruit” among therapeutic targets (77). Importantly, lessons learned regarding the outcome of targeting individual Notch receptors and ligands in cancer-centered clinical trials may guide future therapeutic efforts. Furthermore, developing drug delivery systems for specific cell-targeted inhibition, intermittent administration schedules and dosage optimization have shown promising results and may pave the way from bench to bedside.

**Table 2:** Antibodies against the Notch system used in animal studies.

Name	Isotype	Source	Animal model of inflammation	References *(PMID)*
Anti-Dll1 (YW161.11.7)	IgG1	Genentech, Inc.	GVHD	23454750
Anti-Dll1	Mouse IgG	Juntendo University	RA	22390640
Anti-Dll1 (HMD1-5)	Hamster IgG	Juntendo University	RA, MS	24943093, 17947672
Anti-Dll4 (YW152F)	IgG1	Genentech, Inc.	GVHD	23454750
Anti-Dll4 (HMD4-2)	Hamster IgG	Juntendo University	MS, uveitis, atherosclerosis	20685674 and 21813770, 21896864, 22699504
Anti-Jagged1 (HMJ1-29)	Hamster IgG	Juntendo University	MS*	17947672
Anti-Notch1/NRR1 (YW169.6.0.79)	IgG1	Genentech, Inc.	RA, GVHD	32499639, 23454750
Anti-Notch3/NRR3 (N3.A4)	IgG2a	Genentech, Inc.	RA	32499639

*Anti-Jagged1 antibody treatment exacerbated clinical disease.

## Authors Contributions

PC and TG performed the literature research, wrote, reviewed and formatted the ms. SK contributed to the literature research, wrote and reviewed the ms. GH and JA wrote and reviewed the ms and provided critical scientific input. ES designed the lay out, wrote and reviewed the ms and supervised the study. All authors contributed to the article and approved the submitted version.

## Funding

This research was supported by funds provided by the Research Council of Norway (Grant no. 240814) to ES. Supported in part by the South-Eastern Norway Regional Health Authority (Grant no. 2019059) to PC, by internal funding from Division of Head, Neck and Reconstructive Surgery, Oslo University Hospital and Dr. Jon S Larsens Foundation to TG, and by the Research Council of Norway (Grant no. 287927) and South-Eastern Norway Regional Health Authority (Grant no. 2021069 and 2018052) to JA.

## Conflict of Interest

The authors declare that the research was conducted in the absence of any commercial or financial relationships that could be construed as a potential conflict of interest.

## Abbreviations

ALL, acute lymphoblastic leukemia; AMD, age-related macular degeneration; BMT, bone marrow transplantation; CD, Crohn’s disease; CNS, central nervous system; CNV, choroidal neovascularization; DLL-1, Delta-like 1; DSS, dextran sulfate sodium; EAE, autoimmune encephalomyelitis; EAU, experimental autoimmune uveitis; ECM, extracellular matrix; EGF, epidermal growth factor; GSI, γ-secretase inhibitor; GVHD, graft-versus-host disease; HIF1α, hypoxia-inducible factor 1 alpha; IBD, inflammatory bowel disease; IgG, immunoglobulin G; iNOS, inducible Nitric Oxide Synthase; LDL, low-density lipoprotein; LPS, Lipopolysaccharide; mAbs, monoclonal antibodies; MAML-1, Mastermind-like transcriptional co-activator 1; MCP-1, monocyte chemoattractant protein-1; MS, Multiple sclerosis; NICD, Notch intracellular domain; NRR, negative regulatory region; OPC, oligodendroglial precursor cell; PDGF, platelet-derived growth factor; RA, rheumatoid arthritis; RBP-Jk, Recombination Signal-Binding Protein 1 for J-kappa; SHP-1, Src homology region 2 domain-containing phosphatase-1; SLE, Systemic lupus erythematosus; Ssc, Systemic sclerosis; TACE, tumor necrosis factor-α-converting enzyme; TGFb, transforming growth factor beta; TNF, tumor necrosis factor; TLR, Toll-like receptor; UC, Ulcerative colitis; VEGF, vascular endothelial growth factor; ADC, antibody-drug conjugates; DVD-Ig, dual-variable domain immunoglobulin; HLE BiTE, half-life extended bispecific T cell engager; CAR-T, chimeric antigen receptor T cell; ScFv, single-chain fragment variable; Fab, fragment antigen binding; Fc, fragment crystallizable; FcRn, neonatal Fc receptor.
